# Bites of Reptiles

**Published:** 1880-10-20

**Authors:** J. Hendree

**Affiliations:** Ala.


### BITES OF REPTILES.

BY J. HENDREE, M.D., OF ALA.

I was much interested in the graphic account by Dr. Jones, in your last number, of snake bites. Permit me to state that the best antidote for all reptile and insect poisons is "Eau de Luce," an ammoniacal preparation which ought to be kept in every house, and carried about the person by those exposed to such accidents. Bathe the wound profusely and give a teaspoonful in brandy, or any strong spirit, every ten or fifteen minutes. This makes an active and not disagreeable ammonia julep. The preparation is kept in the Zoological Gardens of Europe. See Frank Buckland's "Zoological Recreations."

Some years since, one of the keepers of the cobras, at Regent's Park, in a state of complete intoxication, entered the cage, and handling one of the serpents roughly and carelessly, was bitten. His intoxication and the profuse use of spirits, without an alkali, did not save him, and he died in a few hours.

The recipe for eau de luce may be had at any druggist's.

